# Sinonasal Chondromyxoid Fibroma: Case Report and Literature Review

**DOI:** 10.7759/cureus.5841

**Published:** 2019-10-05

**Authors:** Nadeem El-Kouri, Alhasan Elghouche, Shaoxiong Chen, Taha Shipchandler, Jonathan Ting

**Affiliations:** 1 Department of Otolaryngology - Head and Neck Surgery, Loyola University Chicago Stritch School of Medicine, Maywood, USA; 2 Department of Medical Education and Simulation, Indiana University School of Medicine, Indianapolis, USA; 3 Department of Pathology and Laboratory Medicine, Indiana University School of Medicine, Indianapolis, USA; 4 Department of Otolaryngology - Head and Neck Surgery, Indiana University School of Medicine, Indianapolis, USA

**Keywords:** chondromyxoid fibroma, cmf, sinonasal tumors, review of chondromyxoid fibroma

## Abstract

Chondromyxoid fibroma (CMF) is a rare, benign neoplasm of the chondroid, myxoid, and fibrous tissue. It characteristically affects the lower extremity long bones, although it may rarely arise within the craniofacial skeleton. We report the diagnosis and management of a 31-year-old male with a large, incidentally discovered CMF originating from the sphenoid sinus. A subsequent review of the literature reveals the need to differentiate from more aggressive neoplasms, such as chondrosarcoma and chondroma, which share radiographic features. A histopathologic examination is crucial for proper diagnosis and treatment. We discuss clinical sequelae, highlight the importance of a thorough pre-operative evaluation, and summarize previously suggested treatment paradigms.

## Introduction and background

Originally described in 1948 by Jaffe and Lichtenstein, the chondromyxoid fibroma (CMF) is a neoplasm of cartilaginous origin [1] accounting for <0.5% of all bone tumors [2-4], with a slight male predominance [5]. Although more commonly found in the metaphysis of long bones, between 1-5% of CMF cases are reported to occur in the head and neck [5-6].

Cases have been reported in all the paranasal sinuses. Given its benign nature, patients may not present until secondary symptoms manifest. These symptoms depend on tumor size and location but may include diplopia, facial pain, exophthalmos, neuralgia, dysarthria, epistaxis, nasal congestion, headache, bony swelling, or persistent chronic rhinosinusitis [7]. Given the rarity of CMF, it is crucial to consider more aggressive neoplasms, including chondrosarcoma and chordoma. We report the diagnosis and management of a patient with CMF originating from the sphenoid sinus and review the available literature describing its presence within the head and neck with a focus on the paranasal sinuses.

Case presentation

**Figure 1:**
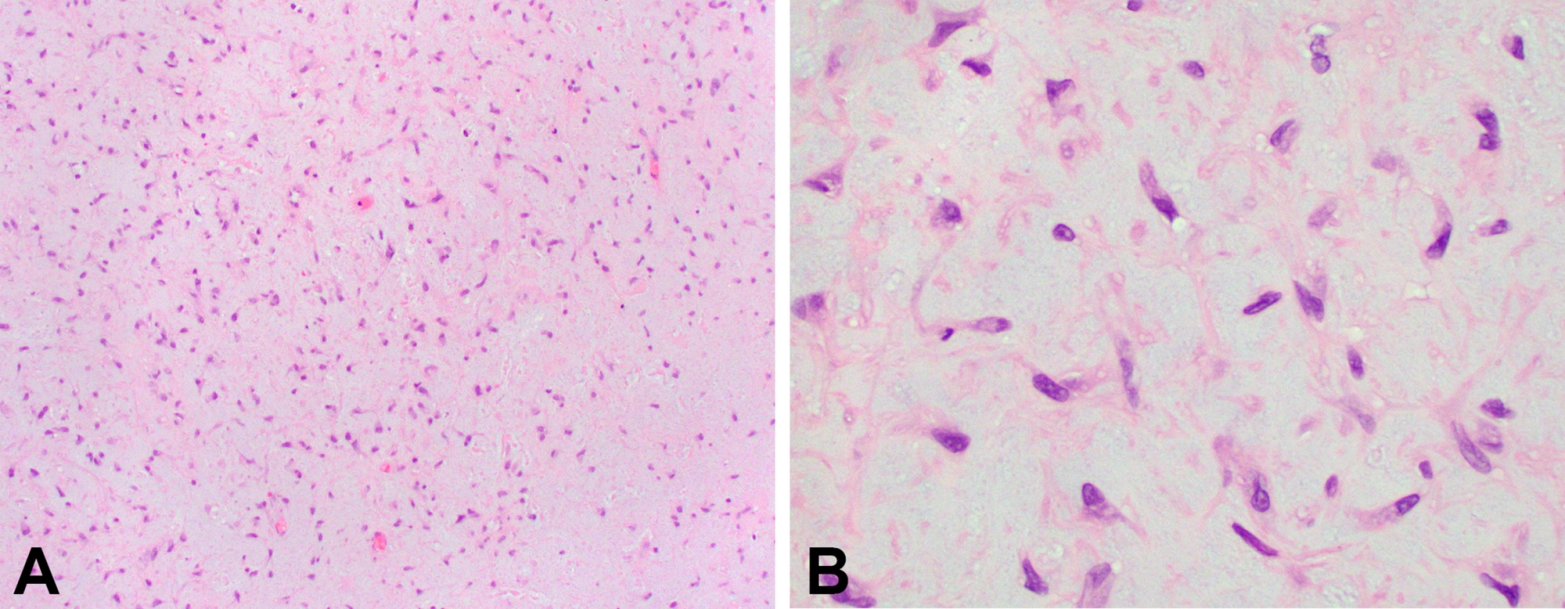
Histopathologic images. A (Left). Microscopic examination reveals stellate or spindle-shaped cells with abundant chondromyxoid matrix (100x). B (Right) Lesional cells with eosinophilic cytoplasm and mildly pleomorphic nuclei containing finely dispersed or homogeneous dark chromatin (400x).

A previously healthy 31-year-old male presented to the Otolaryngology - Head & Neck Surgery clinic, with right-sided nasal airway obstruction. Physical examination, including nasal endoscopy, revealed a large mass within the right nasal passage. He had no associated symptoms such as visual complaints, paresthesia, and facial pain. A biopsy was performed in the operating room, with the histopathologic evaluation revealing a lobular growth pattern with stellate or spindle-shaped cells in a chondroid background (Figure 1). Well-developed hyaline cartilage was not present. The pathology report stated the morphological features were consistent with CMF.

**Figure 2:**
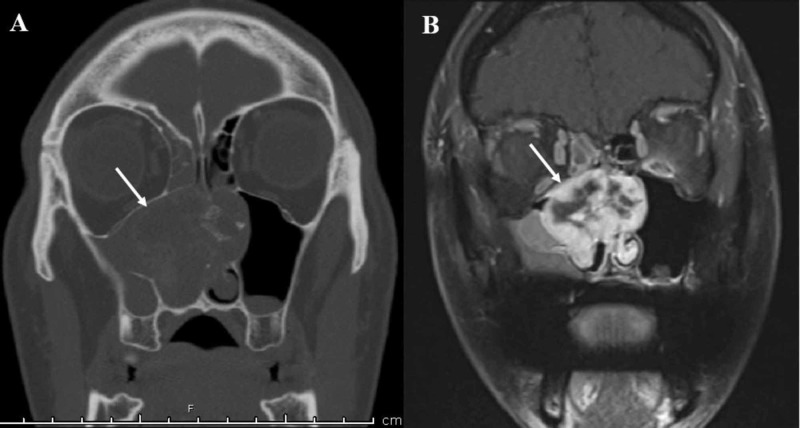
CT and MR imaging. (A) Contrast-enhanced CT scan demonstrating an expansile mass centered within the right nasal cavity with extension into the left nasal passage (white arrow), with scattered calcifications. (B) The same mass (white arrow) observed on contrast-enhanced T1-weighted MRI with heterogeneous uptake.

Prior to definitive resection, preoperative computed tomography (CT) and magnetic resonance imaging (MRI) imaging were completed for anatomic mapping and surgical planning. CT revealed a homogeneously enhancing mass with scattered coarse central calcifications. There was extensive bony remodeling and displacement without aggressive osseous erosion. Although centered within the right nasal cavity, the lesion was found to erode through the nasal septum into the left nasal cavity (Figure 2A). The mass extended to the level of the right nasolacrimal canal anteriorly and to the level of the right choana and nasopharynx inferiorly. Laterally, the mass deformed the medial wall of the left maxillary sinus and obstructed the maxillary sinus ostium with associated sinus opacification. Posterior involvement was noted within the right sphenoid sinus and left posterior ethmoid sinuses. There was no obvious intracranial or intraorbital extension. MRI with and without contrast supported the CT findings and demonstrated cystic components (Figure 2B) with relative T2 hypointensity concerning for high cellularity or a high nuclear-to-cytoplasmic ratio. No perineural spread or adenopathy was noted.

**Figure 3:**
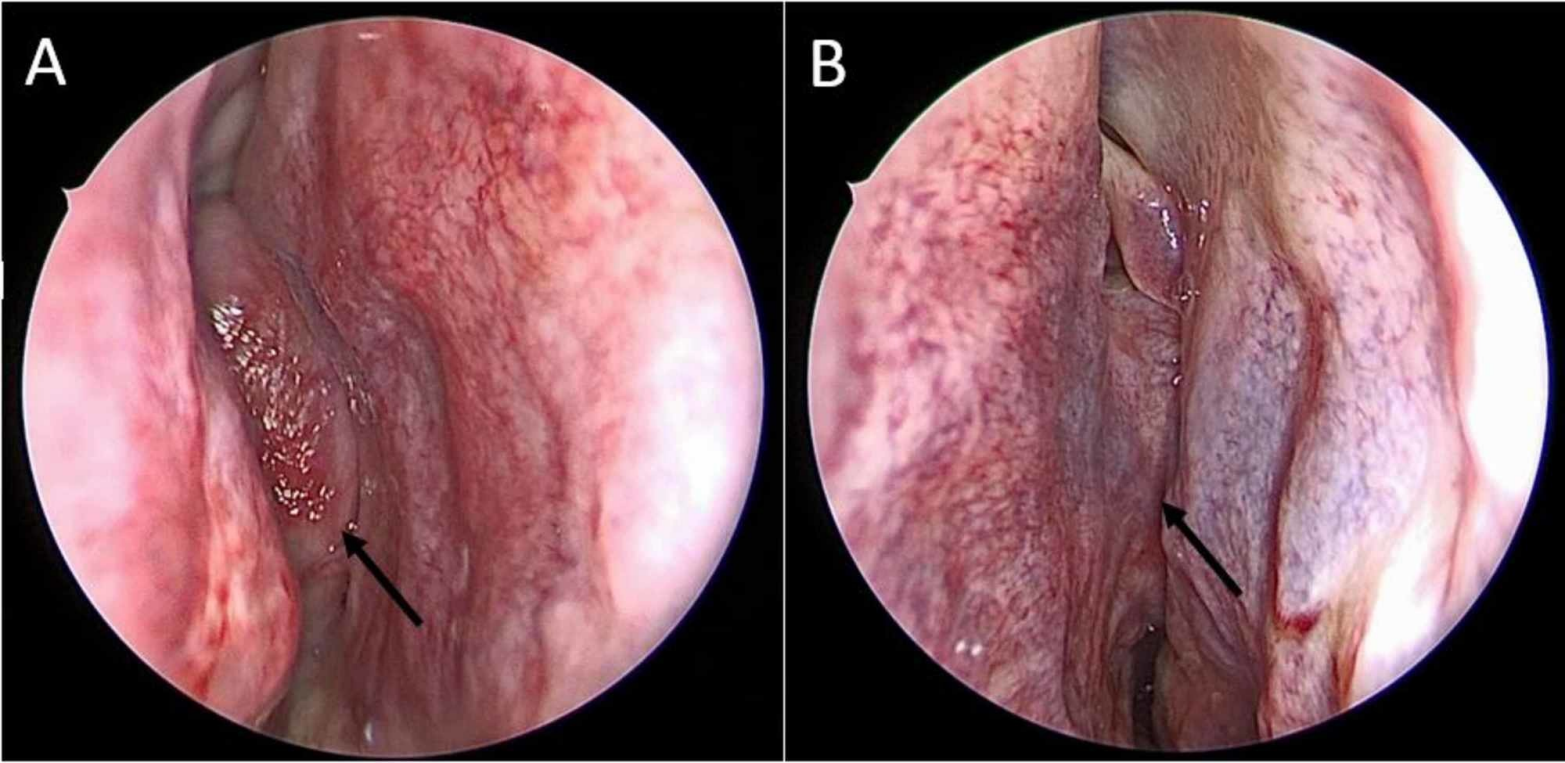
Intraoperative photos. (A) Endoscopic view demonstrating a mass (black arrow) within the right nasal cavity at the level of the middle meatus. (B) Endoscopic view demonstrating extension of the mass (black arrow) across the nasal septum to involve the left nasal cavity.

One month later, the patient underwent endoscopic resection under general anesthesia. Intraoperatively, the tumor was noted to attach to the planum sphenoidale without dural involvement. The tumor was found to extend from the sphenoid with erosion through the posterior septum to involve the left posterior ethmoid cavity (Figure 3).

In addition to complete tumor resection, bilateral maxillary antrostomies and sphenoethmoidectomies were performed as well as a posterior septectomy. The left middle turbinate was also resected due to tumor involvement. There was no intraoperative cerebrospinal (CSF) fluid leak.

**Figure 4:**
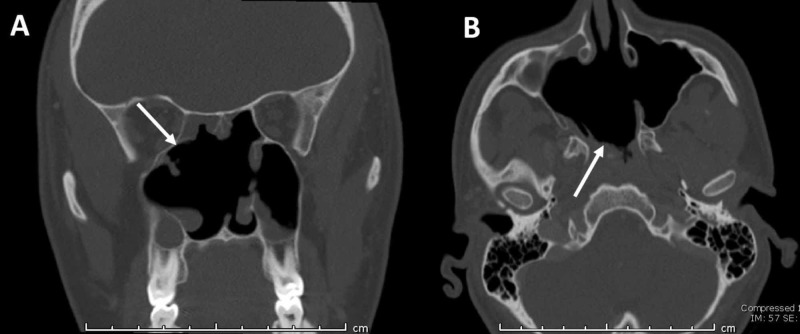
Postoperative CT images. (A) Coronal and (B) axial images showing complete tumor resection (arrows), bilateral maxillary antrostomies and sphenoethmoidectomies and posterior septectomy.

The postoperative course was uneventful and a follow-up CT scan was obtained two weeks later without evidence of residual disease, as seen in Figure 4.

## Review

CMF is a slow-growing and rare neoplasm consisting of chondroid, myxoid, and fibrous tissue. CMF accounts for less than 0.5% of all bone tumors [2-4], with a slight male predominance [5]. Although it is most commonly found in the metaphysis of long bones, craniofacial involvement has been reported and comprised 5.4% of the 278 CMF cases surveyed by Wu et al. [5]. This benign, albeit locally aggressive tumor is most commonly found in patients in the second or third decades of life [8]. The rare occurrence rate of CMF makes it a challenging disease to study directly and the presentation of a single case is a relative limitation of the present study. An examination of the literature reveals a number of studies with reported areas of involvement of much of the skeleton. Within the head and neck, cases have been identified in the paranasal sinuses, mastoid process, sella turcica, and clivus [9]. Additional details, including patient demographic data, location, treatment, and follow-up of all reported cases of paranasal CMF can be found in Table 1.

**Table 1:** Treatment and outcome data for all reported cases of CMF in the paranasal sinuses, arranged by location Abbreviations: DF, disease-free; M, male; F, female; y, year; mo, month; d, day; NA, unable to obtain or not addressed in the article; CMF, chondromyxoid fibroma

Author	Age y, mo, or d/ Sex	Location	Treatment	Follow-up	Clinical presentation	Duration of symptoms
Keel et al. [10]	65 y/F	Clivus with extension to sphenoid and ethmoid sinuses	Curettage and radiation	Local recurrence after 6 mo; after radiation, 20 mo DF	2/3 patients in this series presented with headache while other presented with nasal obstruction	NA
Keel et al. [10]	66 y/F	Clivus with extension to sphenoid and ethmoid sinuses	Curettage	26 mo DF		NA
Isenberg et al. [6]	34 y/F	Ethmoid sinus	Endoscopic excision, ethmoidectomy	8 mo DF	Difficulty breathing	3 y
Mendoza et al. [11]	1 mo/M	Ethmoid sinus	Block resection	2 y DF	Respiratory difficulty	1 mo
Nazeer et al. [12]	20 d/M	Ethmoid sinus	Surgical resection	12 mo DF	Respiratory difficulty since birth	20 d
Szmeja et al. [13]	8 y/F	Ethmoid sinus	Total enucleation	NA	NA	NA
Won et al. [14]	28 y/M	Ethmoid sinus	Partial curettage	NA	Intermittent, pulsatile pain of right temporal area	Long-standing
Cruz et al. [15]	10 y/F	Ethmoid sinus invading orbit	Coronal approach with en bloc resection	NA	Progressive exophthalmos of left eye	7 mo
Hashimoto et al. [16]	32 y/M	Ethmoid sinus, extending to frontal sinus and orbit	Surgical resection	2 y DF	Painless left frontal swelling and progressive exophthalmos	1 y
Azorin et al. [17]	46 y/M	Frontal sinus	Subfrontal approach, superior orbitotomy, and block resection, including pericranium and surrounding frontal bone	22 mo DF	Right supraciliary frontal mass	18 mo
Wolf et al. [18]	35 y/F	Frontal-sphenoid junction with orbital infiltration	Left craniotomy with piecemeal removal	NA	Frontal headache	4 mo
Perez-Fernandez et al. [19]	60 y/M	Maxillary sinus with extension into ethmoid sinus	Endoscopic resection and post-ethmoidectomy, Caldwell-Luc	5 y DF	Left nasal obstruction with recurrent ipsilateral epistaxis	"several months"
Koay et al. [20]	57 y/F	Nasal bone with extension to frontal and ethmoid sinuses	Incomplete surgical excision	NA	Slowly expanding, painless swelling over bridge of nose	2 y
Baujat et al. [21]	50 y/F	Nasal bone, extension into frontal and ethmoidal sinuses with dural involvement	Frontal bone window with dura removal	18 mo DF	Frontal headache, pain, nasal obstruction and tearing	3 mo
Wang et al. [9]	60 y/F	Nasal septum	Complete surgical removal	6 mo DF	No clinical discomfort (MH: congenital right aural atresia)	60 y
Veras et al. [22]	60 y/F	Nasal septum	Surgical excision	12 mo DF	Incidental (asymptomatic)	NA
McClurg et al. [23]	49 y/F	Nasal septum extending into the maxilla	Midface degloving with resection of nasal septum, left ethmoid, and left partial maxilla	16 mo DF	Sinonasal congestion	6 mo
Januszek et al. [24]	51 y/F	Nasal septum, extension into maxillary and sphenoid sinuses	Surgical resection	Recurrence after 12 mo, reoperated	NA	NA
Frank et al. [25]	26 y/M	Petrous/sphenoid bones extending into posterior clinoid, sella, and cavernous sinus	Complete surgical removal	NA	Diplopia	1 mo
Vernon et al. [26]	44 y/M	Sphenoid sinus	Endoscopic sphenoid sinusotomy and posterior ethmoidectomy	NA	Left retroorbital pain	3 mo
Morris et al. [27]	52 y/F	Sphenoid sinus, eroding floor of sphenoid sinus	Endoscopic resection with rim of normal bone	2 y DF	No sinonasal signs or symptoms	NA
Nazeer et al. [12]	66 y/F	Sphenoid sinus, extension into nasopharynx and sella	Surgical curettage	Local recurrence after 1 y, curetted, 6 mo DF	Nasal obstruction	Several years

A review of the literature demonstrates inconsistent presenting symptoms. Table 1 suggests that clinical sequelae arise secondary to the mass effect rather than specific to the acute pathology of CMF. The lack of specificity of symptoms highlights the importance of a thorough examination and workup in obtaining an accurate diagnosis.

Given its rarity, an early differential would seldom, if ever, have CMF as the primary diagnosis. More common lesions, such as a chordoma or chondrosarcoma, should be considered for any lesion demonstrating calcification within a chondroid-like matrix [10]. These neoplasms portend a worse prognosis than CMF, with 10-year survival rates of 50% for skull base chondrosarcomas and 16-32% for skull base chordomas [28].

These lesions are also difficult to differentiate radiologically. The identification of calcifications on CT should prompt the consideration of a cartilaginous or chondroid matrix-containing lesion [23]. Although some CMF lesions contain foci of calcification, these are frequently microscopic and, therefore, rarely visible on imaging [29]. Chondrosarcomas, by contrast, contain prominent, easily seen calcifications [23]. Of the 191 cases of CMF that Wu et al. studied, 87% had a purely lucent matrix while 15% had signs of mineralization [5]. MRI is the preferred radiologic modality to understand the extent of the disease process, as it provides information on soft tissue involvement. On MRI, these three lesions are all hypointense or isointense on T1 and hyperintense on T2, with frequent enhancement via gadolinium contrast, if used.

Given this overlap of imaging findings across various lesions, a detailed histopathologic evaluation becomes crucial in making the proper diagnosis. In their series of 36 patients with CMF, Zilmer and Dorfman reported a 22% initial misdiagnosis rate and pointed out the potential of more aggressive treatment than is required, such as amputating the affected limb for a benign lesion [30-31]. The World Health Organization defines CMF as “a benign tumor characterized by lobules of spindle-shaped or stellate cells with abundant myxoid or chondroid intercellular material separated by zones of more cellular tissue rich in spindle-shaped or round cells with a varying number of multinucleated giant cells of different sizes’’ [32]. Stellate or spindle-shaped cells have centrally located nuclei with finely dispersed or homogeneous dark chromatin and eosinophilic cytoplasm. Nuclear pleomorphism and atypia are uncommon in CMF and are more defining of chondrosarcoma [15,31,33]. Additionally, the intersecting bands of myxochondroid tissue are not found in chondrosarcoma. According to Castle et al., diagnostic features of chondrosarcomas also include a “bubbly appearance to the stroma along with degenerative and liquefactive changes” [31]. Chordomas, in contrast, have the characteristic physaliferous cells (those with abundant, bubbly, or vacuolated eosinophilic cytoplasm), which are nearly pathognomonic for the lesion [27].

Although the standard of treatment for CMF is surgical excision, the cosmetic and functional consequences of extensive resection of craniofacial lesions have led many authors to recommend curettage specifically for facial CMF with close follow-up. Baujat et al. asserted that long-term control is best achieved via wide surgical resection by demonstrating the high recurrence rate achieved after curettage [21]. Select cases, such as the one described above, may be amenable to endoscopic resection.

Another reason to favor surgery involves the risk of residual tissue undergoing malignant transformation. Although the transformation rate is low [34], cases of malignant transformation have been reported, with an increased risk noted after radiation therapy [4,30,35]. A location at or near the skull base, however, imposes additional difficulty in obtaining complete resection [10]. As such, some authors have suggested postoperative irradiation as an attempt to reduce recurrence [36].

## Conclusions

A review of the literature demonstrates that although an exceedingly rare tumor, representing <0.5% of all bone tumors, CMF has been reported to occur in the sinonasal cavity. Although CMF may appear similar to chondrosarcoma and chordoma on imaging, proper treatment depends on histopathologic differentiation. A biopsy will reveal stellate cells within an abundance of a myxoid or chondroid matrix. Although curettage may be necessary in the case of inaccessible tumors, complete surgical resection is preferred to minimize the risk of recurrence.
